# Pediatric Renal Transplantation in a Highly Sensitised Child—8 Years On

**DOI:** 10.1155/2011/370596

**Published:** 2012-01-26

**Authors:** Catherine Quinlan, Atif Awan, Denis Gill, Mary Waldron, Dilly Little, David Hickey, Peter Conlon, Mary Keogan

**Affiliations:** ^1^Paediatric Renal Transplant Centre, The Children's University Hospital, Temple Street, Dublin 1, Ireland; ^2^Our Lady's Children's Hospital, Crumlin, Dublin 12, Ireland; ^3^Renal Transplant Unit, Beaumont Hospital, Dublin 9, Ireland; ^4^National Histocompatibility and Immunogenetics Services for Solid Organ Transplantation, Beaumont Hospital, Dublin 9, Ireland

## Abstract

Highly sensitised children have markedly reduced chances of receiving a successful deceased donor renal transplant, increased risk of rejection, and decreased graft survival. There is limited experience with the long-term followup of children who have undergone desensitization. Following 2 failed transplants, our patient was highly sensitised. She had some immunological response to intravenous immunoglobulin (IVIg) but this was not sustained. We developed a protocol involving sequential therapies with rituximab, IVIg, and plasma exchange. Immunosuppressant therapy at transplantation consisted of basiliximab, tacrolimus, mycophenolate mofetil, and steroids. At the time of transplantation, historical crossmatch was ignored. Current CDC crossmatch was negative, but T and B cell flow crossmatch was positive, due to donor-specific HLA Class I antibodies. Further plasma exchange and immunoglobulin therapy were given pre- and postoperatively. Our patient received a deceased donor-kidney-bearing HLA antigens to which she originally had antibodies, which would have precluded transplant. The graft kidney continues to function well 8 years posttransplant.

## 1. Introduction

Kidney transplantation remains the optimal treatment for end-stage renal failure (ESRF). However, highly sensitised children continue to have markedly reduced chances of receiving a successful kidney transplant and, if transplanted, they have a greater number of rejection episodes with decreased graft survival [1]. Over the last 10–15 years, there has been increased interest in transplanting sensitised children, due to increased rates of living-donor transplants, better immunosuppressive regimes, improved methods of detecting anti-HLA antibodies, and a greater understanding of antibody-mediated rejection (AMR) [2].

Several protocols have been suggested for desensitization of patients who have a positive cross-match against a potential donor, utilizing a combination of IVIg [3–5], plasmapheresis [6], MMF [7], sirolimus [8], and rituximab [9–15]. There is limited data on desensitization facilitating deceased donor transplantation and little long-term followup data. We describe a patient who underwent successful deceased donor transplantation 7 years ago using a hybrid of the John Hopkins [6] and Cedars Sinai [5] desensitization protocols.

## 2. Patient

Our patient was a 12-year-old Caucasian girl with ESRF secondary to congenital nephrotic syndrome of the Finnish variety [16]. She had renal transplants at age 3 and 5 years. The first was lost to chronic allograft nephropathy at 18 months. The second was lost to acute rejection necessitating nephrectomy, within 2 weeks of transplantation. During the transplant nephrectomy, blood loss necessitated resuscitation with 7 units of red cells. Her first allograft remained in situ.

She was then maintained on hemodialysis for 8 years without a suitable kidney becoming available. Her antibody levels remained high with a panel reactive antibody (PRA) determined by Complement Dependent Lymphocytotoxic (CDC) of 95% against peripheral blood lymphocytes and 100% against chronic lymphocytic leukaemia (CLL) cells. No suitable living donor was available. After 60 months on the waiting list for a deceased donor transplant, the decision was made to attempt desensitization. After failure of IVIg on its own to desensitize her, a novel regime was developed as outlined below.

## 3. Desensitization Regime

### 3.1. Pretransplant

- Rituximab (375 mg/m^2^), four infusions administered weekly.
- Single volume plasma exchange with fresh frozen plasma (FFP) replacement monthly for 3 months.
- Infusion of IVIg (flebogamma 2 g/kg over 2 days) monthly for 3 months after each plasma exchange.

### 3.2. Immediately Pretransplant

- Plasma exchange with FFP replacement.
- Infusion of IVIg (flebogamma 500 mg/kg).

### 3.3. Immediately Posttransplant (Week 1)

- Plasma exchange with 4.5% human albumin replacement.
- Infusion of IVIg (500 mg/kg) daily for 4 days on day 3, 4, 5, and 6.
- Infusion of rituximab (375 mg/m^2^).

## 4. Immunosuppression Regime

### 4.1. Pretransplant

- Basiliximab 10 mg IV 2 hours pretransplant.
- Prednisolone 600 mg/m^2^ IV.
- Tacrolimus 0.15 mg/kg IV.

### 4.2. Posttransplant

- Mycophenolate mofitil 250 mg twice daily (14 mg/kg/day).
- Prednisolone tapered to 10 mg/day by day 7 posttransplant.
- Basiliximab 10 mg IV day 4 posttransplant.
- Tacrolimus 0.3 mg/kg/day post transplant adjusted with serum drug levels (aim 12–15 in first 3 months).

Our patient received a blood group identical, HLA 1,1,2 mismatch kidney from an unrelated, standard criteria, deceased donor. CDC crossmatch was negative, but flow cytometry crossmatch was significantly positive against both T and B cells. Subsequent investigation of the day of transplant serum showed donor-specific antibodies against A31 and borderline reactivity to DQ2. The A31 and DQ2 antibodies were presumed to be due to transfusion, while the historic reactivity to DR53 was related to previous mismatches.

Donor-specific antibodies were monitored by flow cytometry using donor cells or ELISA thrice weekly for 3 weeks, weekly for 6 weeks, and monthly thereafter. Donor-specific antibodies were noted to be mildly raised, day 3 posttransplant, and this was treated with plasma exchange and further doses of IVIg (total dose 2 g/kg) on day 3, 4, 5, and 6 as outlined above. Donor-specific antibodies were no longer detectable by ELISA. Retrospective reanalysis of stored sera using Luminex single antigen technology has shown persisting donor-specific weak positivity against HLA A31 and DQ2, however, this has remained stable over the last 6.5 years.

She is now 8 years post transplant and has had no episodes of rejection. Her most recent estimated glomerular filtration rate (eGFR) is 71 mL/minute/1.73 m^2^ with a serum creatinine of 104 *μ*mol/L.

## 5. Discussion

Sensitizing events include previous transplantation, blood transfusions, and pregnancy [17]. As many HLA antigens are closely related, exposure to a small number of HLA antigens can give rise to broad sensitization in individuals who generate antibodies to “public,” or cross-reactive, epitopes. Desensitization is an attempt to firstly remove anti-HLA immunoglobulin (IgG), transplant when crossmatch is negative or acceptable, and finally to prevent formation of further donor-specific anti-HLA IgG after transplantation.

At the time of transplantation, 10% of patients on the renal transplant deceased donor waiting list have >60% panel reactive antibody (PRA) levels to HLA [18]. A high PRA limits the number of negative crossmatches with potential donors, thus the waiting time for a suitable kidney is increased. In the past, the presence of these preformed anti-HLA donor specific antibodies (DSA) in the recipient's serum at the time of transplantation was a contraindication to proceeding [2, 19–22]. However, in the last 15 years, there has been increased research in the area of desensitization and a number of approaches have emerged [6, 23, 24].

The cautious and cost-effective approach is to firstly maximise the chance of their receiving negative crossmatch grafts. This can be achieved through acceptable mismatch programs [25] and living donor kidney exchange programs [7]. Results from the Eurotransplant Acceptable Mismatch Program [26] are encouraging, no additional immunosuppression is necessary and graft survival in the sensitised group is identical to that of nonsensitised recipients. The Dutch National Living Donor Kidney Exchange Program [27], The New England Program for Kidney Exchange, the Mid-Atlantic Paired Exchange Program [7], and computer match programs for paired kidney exchanges [28, 29] also offer effective strategies to expand the donor pool. Much work is needed to fund and build such programs at national, European, and international levels.

IVIg is known to have immunomodulatory effects [3, 4], when used as part of a desensitization protocol, it results in decreased anti-HLA antibody levels, decreased ischemia reperfusion injuries, decreased episodes of acute rejection, and improved long-term survival of the graft [8, 30, 31]. It is the only element of the desensitization protocol that has been evaluated in a placebo-controlled, multicentre, double-blinded trial [32]. The NIH IG02 trial enrolled 101 highly sensitised patients with ESRF over 3 years. Patients in the treatment group were given high-dose IVIg (2 g/kg) monthly for 4 months and had significantly lower anti-HLA antibody levels and greater transplantation rates when compared to the placebo group (35% versus 17%  *P* = 0.02). 3-year graft survival rate was not significantly affected.

Plasmapheresis has been used with success in an *ex vivo* model of xenograft hyperacute rejection [33], in patients transplanted across ABO blood groups [34, 35], in heart transplant patients with acute rejection [36], and as part of the pretransplantation management of adult sensitised patients [37–40]. It is used along with cytomegalovirus immunoglobulin as part of the John Hopkins desensitization protocol [6].

The increased understanding of the role played by B-cells in rejection led to the use of agents which specifically target this facet of the immune response [9]. One such agent is rituximab, a genetically engineered chimeric mouse/human monoclonal antibody [10, 11] directed against the CD20 antigen found on the surface of B-lymphocytes. Rituximab eliminates B cells by a combination of antibody-dependent cell-mediated cytotoxicity [12], complement-dependent cytotoxicity [13], and activation of apoptotic pathways [14]. Phase 1 trials of rituximab in nine highly sensitised adults on dialysis showed prolonged depletion of B cells and reductions in PRA [15]. However, rituximab has no effect on plasma cells and no immediate effect on circulating antibody levels, thus it is better used in combination therapy (i.e., with IVIG ± plasma exchange).

We developed a regime which involved sequential therapies with rituximab, IVIg, and plasma exchange. This resulted in a marked sustained reduction in antibody levels, allowing transplantation. Our patient remains well, 102 months posttransplant. She has been successfully transitioned to adult services. Immunosuppression is maintained on MMF and tacrolimus. Her most recent estimated glomerular filtration rate (eGFR) is 71 mL/minute/1.73 m^2^ with a serum creatinine of 104 *μ*mol/L.
